# Breastfeeding duration modified the effects of neonatal and familial risk factors on childhood asthma and allergy: a population-based study

**DOI:** 10.1186/s12931-021-01644-9

**Published:** 2021-02-06

**Authors:** Yabin Hu, Yiting Chen, Shijian Liu, Fan Jiang, Meiqin Wu, Chonghuai Yan, Jianguo Tan, Guangjun Yu, Yi Hu, Yong Yin, Jiajie Qu, Shenghui Li, Shilu Tong

**Affiliations:** 1 Department of Clinical Epidemiology and Biostatistics, Shanghai Children’s Medical Center, Shanghai Jiao Tong University School of Medicine, 1678 Dongfang Road, Pudong, Shanghai, 200127 China; 2 School of Public Health, Shanghai Jiao Tong University School of Medicine, 227 South Chongqing Road, Huangpu, Shanghai, 200025 China; 3 Department of Developmental and Behavioral Pediatrics, Pediatric Translational Medicine Institution, Shanghai Children’s Medical Center, Shanghai Jiao Tong University School of Medicine, Shanghai, China; 4 Shanghai Key Laboratory of Environmental and Child Health, Xinhua Hospital, Shanghai Jiao Tong University School of Medicine, Shanghai, China; 5 Shanghai Key Laboratory of Meteorology and Health (Shanghai Meteorological Service), Shanghai, China; 6 Center for Biomedical Informatics, Shanghai Children’s Hospital, Shanghai, China; 7 Department of Respiratory Medicine, Shanghai Children’s Medical Center, Shanghai Jiao Tong University School of Medicine, Shanghai, China; 8 Shanghai Municipal Education Commission, Shanghai, China; 9 School of Public Health, Institute of Environment and Population Health, Anhui Medical University, Hefei, China; 10 Center for Global Health, School of Public Health, Nanjing Medical University, Nanjing, China; 11 School of Public Health and Social Work, Institute of Health and Biomedical Innovation, Queensland University of Technology, Brisbane, Australia

**Keywords:** Asthma, Allergic disease, Association, Breastfeeding, Children

## Abstract

**Background:**

Childhood asthma and allergic diseases are a significant global problem. There are inconsistent findings on the associations of delivery mode, the number of children in the household and breastfeeding with childhood asthma and allergic diseases. We assessed these associations and examined whether breastfeeding modified the effects of neonatal and familial risk factors on childhood asthma and allergic diseases.

**Methods:**

A population-based cross-sectional study was conducted in Shanghai, China. A total of 17 primary schools were randomly selected from 13 districts of Shanghai in this study. The International Study of Asthma and Allergies in Childhood questionnaire was adopted to assess the childhood asthma and allergic diseases. Multivariable logistic regression models were used to evaluate the associations between neonatal and familial factors and childhood asthma and allergic diseases, and to examine the modification effects of breastfeeding on the associations assessed.

**Results:**

Of 10,464 primary school children aged 6–11 years, the overall prevalence of childhood asthma, allergic rhinitis, urticaria, food allergy and drug allergy was 13.9, 22.7, 15.3, 8.1 and 4.6%, respectively. Male sex, high socioeconomic status, cesarean section delivery, only one child in the household and having family history of allergy were associated with increased odds ratio (OR) of childhood asthma and allergic diseases while longer breastfeeding duration (> 6 months) was inversely associated with these diseases. Longer breastfeeding duration also attenuated the OR of neonatal and familial risk factors on childhood asthma and allergic diseases. For instance, the adjusted OR of childhood asthma in the group of vaginal delivery and breastfeeding duration > 6 months was lowest (0.78, 95% confidence interval: 0.66, 0.92).

**Conclusions:**

Longer breastfeeding duration was inversely associated with childhood asthma and allergic diseases, and also reduced the OR of neonatal and familial risk factors on these diseases. Giving the prevalence of childhood asthma and allergic diseases is rapidly rising across the globe, these findings may have important clinical and public health implications.

## Introduction

The prevalence of childhood asthma and allergic diseases has elevated worldwide in the past 3 decades. For instance, the International Study of Asthma and Allergies in Childhood (ISAAC) phase 3 showed a significant increase in the prevalence of childhood asthma and allergic rhinitis (AR) in most of Asia–Pacific region from 2001 to 2010 [1]. With population and economic growth and urbanization, China has been experiencing a rapid increase in the prevalence of childhood asthma and allergic diseases [2–5]. These changes have brought heavy economic burdens to individuals, families and the society as a whole.

Mounting evidence suggests that prenatal and neonatal factors are associated with the development of childhood asthma and allergic diseases [6–8]. In China, a study conducted in Guangzhou, China found that male sex, high birth weight, cesarean section (CS) delivery, and only one child in the household were associated with the risks of allergic diseases in children aged 6–18 years [8]. Another study in Shanghai found that CS without medical indication was associated with increased risks of both childhood asthma and AR, and exclusive breastfeeding in the first six months attenuated these risks [6]. The Allergic Rhinitis Cohort Study for kids conducted in South Korea found that long-term breastfeeding (≥ 12 months) and a vaginal delivery were associated with a lower risk of developing childhood AR [7]. However, in two unselected populations using harmonised protocols in Australia, the association of CS with developing childhood allergy was weak, and no modification effects by breastfeeding duration were found [9].

Knowledge gaps exist in the associations between prenatal and neonatal factors and development of childhood asthma and allergic diseases. To assess these associations, and to examine whether breastfeeding duration modifies these associations, we conduct a population-based cross-sectional study among primary school children aged 6–11 years in Shanghai, the largest metropolitan city in China.

## Materials and methods

### Study participants

Shanghai is located in the Yangtze River Delta region, east of China, E 120°52′-122°12′, N 30°40′-31°53′, which has over 27 million residents in 2020 [10]. A city-wide cross-sectional survey was conducted during April-June 2019 across Shanghai. To obtain a representative sample, a multi-stage and multi-strata sampling approach was used. Six urban districts (Huangpu, Hongkou, Changning, Xuhui, Putuo, and Yangpu) and seven suburban/rural districts (Pudong, Minhang, Jinshan, Songjiang, Qingpu, Baoshan, and Chongming) were randomly selected using a random number method from 16 districts of Shanghai (7 urban districts and 9 suburban/rural districts). Then, a total of 17 primary schools were randomly selected. As this project is funded by Shanghai Municipal Government, we have gained strong support from all selected schools. According to the ethical principles, study subjects should be informed of the purpose and content of the project. Before the epidemiological survey, we had a meeting with all the principals and head teachers of selected schools and informed them of the details of the project (e.g., the purpose, content and procedure of the project). Then, the principal and the head teacher of each school informed the parents to let them fully understand and cooperate with this research. For the data collection, almost all mothers of the sampled students completed the questionnaires. Overall, 10,700 participants completed the questionnaires. After the exclusion of some children with inappropriate ages (e.g. < 6 years or > 12 years), 10,464 (97.8%) primary school children aged 6–11 years were finally included in this study.

The ethical application and the consent procedure of this study were approved by the Ethics Committees of Shanghai Jiao Tong University School of Medicine and Shanghai Children’s Medical Center.

### Questionnaire

The questionnaire included questions on childhood asthma and allergic diseases, general characteristics of children and parents, socioeconomic status (SES), allergy history of children and their family members, exposure during pregnancy, children’s lifestyle habits, and home environmental exposures in early life. The Cronbach's alpha coefficient of the ISAAC allergic questionnaire in our sampled children was 0.91. The intra-class correlation coefficient of retest reliability at intervals of 2 weeks was 0.94. Validity presented by Kaiser–Meyer–Olkin test was 0.94. These results show the high validity of the questionnaire for the primary school children.

#### Assessment of asthma and allergic diseases

The core question of the ISAAC questionnaire was applied to assess asthma and allergic diseases status [11]. Asthma: Has your child ever been diagnosed with asthma by a doctor? A similar question was asked for AR, urticaria and drug allergy (DA). For food allergy (FA), the question was: Whether your child has a history of FA (red lips, rash, abdominal pain, etc. within 2 h after eating)?

#### Assessment of independent variables

Mode of delivery included vaginal delivery (VD) and caesarean section (CS). Breastfeeding duration was divided into two groups according to WHO recommendations. One was exclusive breastfeeding duration ≤ 6 months, and another was exclusive breastfeeding for 6 months, and continued breast feeding (breastfeeding duration > 6 months). The number of children in household was categorized as: only one child or not. A question about family history of allergy was: Does anyone at home have the following allergic diseases (Asthma, allergic dermatitis, FA, DA, AR, anaphylactic shock, etc.)? Socioeconomic status (SES) was measured as: Do you own the apartment or not?

### Statistical analysis

Statistical descriptions were made utilizing the frequency and percentage for categorical variables. Pearson’s chi-squared (*χ*^2^) test was used to assess the differences among the prevalence of asthma and allergic diseases between different groups. Univariate logistic regression model was used to calculate the unadjusted odds ratios (OR) and 95% confidence interval (CI) of child’s sex, age, SES, mode of delivery, breastfeeding duration, only one child in the household and family history of allergy. Then, multivariable logistic regression model was performed to calculate adjusted OR (AOR) and 95% CI after adjustment for putative confounders. Furthermore, the modification effects of breastfeeding duration on the associations between neonatal and familial factors and children’s allergic diseases were assessed using stratified analyses. All statistical analyses were conducted using R (version 3.6.3; R Core Team).

## Results

In this study, the overall prevalence of childhood asthma, AR, urticaria, FA and DA was 13.9, 22.7, 15.3, 8.1 and 4.6%, respectively. Table 1 shows the summary statistics of characteristics among 10,464 primary school children aged 6–11 years, and the prevalence rates of asthma and allergic diseases among different groups. This study included 5464 (52.2%) boys and 5000 (47.8%) girls, with higher prevalence of asthma and allergic diseases in boys than girls. Average age was 9.2 (standard deviation = 2.2) years. The prevalence of asthma, AR, urticaria and FA was significantly higher in CS group than VD group. The prevalence of asthma, AR, urticaria and DA was significantly lower in the group of breastfeeding duration > 6 months, compared to that in the group of breastfeeding duration ≤ 6 months. The prevalence of childhood asthma and allergic diseases significantly increased (*p* < 0.001) in the group with higher SES (having the home ownership) or with family history of allergy or with only one child in the household. The prevalence of childhood asthma and allergic diseases was significantly higher in urban or suburban area group compared with the group of rural.

**Table 1:** Summary statistics of characteristics and the prevalence of asthma and allergic diseases among different groups

Variable	Total (%)	Asthma (%)	*p*-value	AR (%)	*p*-value	Urticaria (%)	*p*-value	FA (%)	*p*-value	DA (%)	*p*-value
Prevalence	10,464	1453 (13.9)		2372 (22.7)		1600 (15.3)		851 (8.1)		477 (4.6)	
Sex			< 0.001		< 0.001		0.010		0.091		0.031
Boys	5464 (52.2)	883 (16.2)		1429 (26.2)		883 (16.2)		468 (8.6)		272 (5.0)	
Girls	5000 (47.8)	570 (11.4)		943 (18.9)		717 (14.3)		383 (7.7)		205 (4.1)	
Age (year)			0.251		0.447		0.071		0.740		0.166
6	713 (6.8)	103 (14.4)		145 (20.3)		118 (16.5)		63 (8.8)		31 (4.3)	
7	2138 (20.4)	290 (13.6)		469 (21.9)		360 (16.8)		184 (8.6)		86 (4.0)	
8	2092 (20.0)	320 (15.3)		498 (23.8)		323 (15.4)		174 (8.3)		85 (4.1)	
9	2045 (19.5)	289 (14.1)		459 (22.4)		277 (13.5)		160 (7.8)		90 (4.4)	
10	1988 (19.0)	253 (12.7)		459 (23.1)		304 (15.3)		148 (7.4)		102 (5.1)	
11	1488 (14.2)	198 (13.3)		342 (23.0)		218 (14.7)		122 (8.2)		83 (5.6)	
Mode of delivery			< 0.001		< 0.001		< 0.001		0.010		0.094
Vaginal delivery	4224 (40.4)	523 (12.4)		878 (20.8)		576 (13.6)		308 (7.3)		175 (4.1)	
Caesarean section	6240 (59.6)	930 (14.9)		1494 (23.9)		1024 (16.4)		543 (8.7)		3002 (4.8)	
Breastfeeding duration (months)			< 0.001		< 0.001		0.008		0.132		0.001
≤ 6 m	5373 (51.3)	825 (15.4)		1388 (25.8)		870 (16.2)		458 (8.5)		279 (5.2)	
> 6 m	5091 (48.7)	628 (12.3)		984 (19.3)		730 (14.3)		393 (7.7)		198 (3.9)	
Birth weight (g)			0.388		0.569		0.593		0.837		0.737
< 2500 g	298 (3.0)	50 (16.8)		62 (20.8)		52 (17.4)		26 (8.7)		11 (3.7)	
2500–4000 g	8896 (88.5)	1251 (14.1)		2054 (23.1)		1379 (15.5)		726 (8.2)		414 (4.7)	
≥ 4000 g	855 (8.5)	117 (13.7)		190 (22.2)		128 (15.0)		74 (8.7)		40 (4.7)	
Gestational week (weeks)			0.027		0.570		0.406		0.213		0.399
< 37w	946 (9.1)	158 (16.7)		222 (23.5)		159 (16.8)		91 (9.6)		43 (4.5)	
37-42w	8972 (86.6)	1219 (13.6)		2039 (22.7)		1370 (15.3)		722 (8.0)		406 (4.5)	
≥ 43w	440 (4.2)	65 (14.8)		92 (20.9)		64 (14.5)		33 (7.5)		26 (5.9)	
Only child			< 0.001		< 0.001		< 0.001		< 0.001		< 0.001
Yes	6524 (62.3)	990 (15.2)		1687 (25.9)		1104 (16.9)		590 (9.0)		347 (5.3)	
No	3940 (37.3)	463 (11.8)		685 (17.4)		496 (12.6)		261 (6.6)		130 (3.3)	
Family historyof allergy			< 0.001		< 0.001		< 0.001		< 0.001		< 0.001
Yes	2450 (23.4)	639 (26.1)		1094 (44.7)		636 (26.0)		412 (16.8)		205 (8.4)	
No	8014 (76.6)	814 (10.2)		1278 (15.9)		964 (12.0)		439 (5.5)		272 (3.4)	
Home ownership			< 0.001		< 0.001		< 0.001		< 0.001		< 0.001
Have	7743 (74.0)	1179 (15.2)		2015 (26.0)		1345 (17.4)		701 (9.1)		391 (5.0)	
Not have	2721 (26.0)	274 (10.1)		357 (13.1)		255 (9.4)		150 (5.5)		86 (3.2)	
Residential area			0.013		< 0.001		< 0.001		< 0.001		0.014
Urban	5324 (50.9)	756 (14.2)		1367 (25.7)		887 (16.7)		481 (9.0)		256 (4.8)	
Urban–rural juction	4077 (39.0)	584 (14.3)		855 (21.0)		599 (14.7)		316 (7.8)		192 (4.7)	
Industrial zone	79 (0.8)	10 (12.7)		9 (11.4)		10 (12.7)		6 (7.6)		0 (0.0)	
Rural	984 (9.4)	103 (10.5)		141 (14.3)		104 (10.6)		48 (4.9)		29 (2.9)	

Data were presented with frequency and percentage; *p*-value was calculated by Pearson’s chi-squared (*χ*^2^) test;

Univariate and multivariate logistic regression analyses between childhood allergy and independent variables (Table 2) suggest that boys, higher SES, CS delivery, only one child in the household and having family history of allergy increased the OR of childhood allergy, while longer breastfeeding duration (> 6 months) decreased the OR of childhood allergy. For instance, the AOR of childhood asthma was 1.53 (95% CI: 1.36, 1.72) for boys, 1.27 (95% CI: 1.09, 1.47) for higher SES, 1.16 (95% CI: 1.03, 1.32) for only one child in the household, 2.99 (95% CI: 2.65, 3.36) for having family history of allergy and 0.84 (95% CI: 0.75, 0.95) for breastfeeding duration > 6 months. We also did sensitivity analysis of multivariate logistic regression after adjustment for residential area and whether the address changed after birth (Additional file 1: Table S1). The results were almost the same with that of Table 2.

**Table 2:** Results of univariate and multivariate logistics regression analyses between childhood allergy and independent variables

Variable	Asthma		Allergic rhinitis		Urticaria		Food Allergy		Drug allergy	
	COR	AOR	COR	AOR	COR	AOR	COR	AOR	COR	AOR
Sex(girls)										
Boys	*1.50 (1.34,1.68)*	*1.53 (1.36,1.72)*	*1.52 (1.39,1.67)*	*1.62 (1.46,1.78)*	*1.15 (1.04,1.28)*	*1.17 (1.05,1.30)*	1.13 (0.98,1.30)	1.13 (0.98,1.31)	*1.23 (1.02,1.48)*	*1.23 (1.02,1.48)*
Age	0.98 (0.94,1.02)	0.99 (0.95,1.02)	1.02 (0.99,1.05)	1.03 (1.00,1.07)	*0.96 (0.93,1.00)*	0.97 (0.93,1.00)	0.97 (0.92,1.01)	0.98 (0.93,1.03)	*1.09 (1.02,1.16)*	*1.09 (1.03,1.17)*
Home ownership (Not have)										
Have	*1.60 (1.40,1.84)*	*1.27 (1.09,1.47)*	*2.33 (2.06,2.63)*	*1.76 (1.54,2.01)*	*2.03 (1.76,2.34)*	*1.68 (1.45,1.95)*	*1.71 (1.42,2.05)*	*1.31 (1.08,1.59)*	*1.63 (1.29,2.07)*	1.22 (0.95,1.57)
Mode of delivery (VD)										
CS	1.24 (1.11,1.39)	1.12 (0.99,1.26)	*1.20 (1.09,1.32)*	1.00 (0.91,1.11)	*1.24 (1.11,1.39)*	1.12 (1.00,1.25)	*1.21 (1.05,1.40)*	1.10 (0.95,1.28)	1.18 (0.97,1.42)	1.02 (0.84,1.24)
Breastfeeding duration (≤ 6 m)										
> 6 m	*0.78 (0.69,0.87)*	*0.84 (0.75,0.95)*	*0.69 (0.63,0.76)*	*0.77 (0.70,0.85)*	*0.87 (0.78,0.96)*	0.97 (0.87,1.08)	0.90 (0.78,1.03)	0.99 (0.86,1.15)	*0.74 (0.61,0.89)*	*0.82 (0.68,0.99)*
Only child (No)										
Yes	*1.34 (1.19,1.51)*	*1.16 (1.03,1.32)*	*1.66 (1.50,1.83)*	*1.37 (1.23,1.53)*	*1.41 (1.26,1.59)*	*1.19 (1.05,1.34)*	*1.40 (1.21,1.63)*	*1.22 (1.04,1.43)*	*1.65 (1.34,2.02)*	*1.46 (1.18,1.80)*
Family historyof allergy (No)										
Yes	*3.12 (2.78,3.50)*	*2.99 (2.65,3.36)*	*4.25 (3.85,4.70)*	*4.02 (3.63,4.45)*	*2.56 (2.29,2.87)*	*2.37 (2.12,2.66)*	*3.49 (3.02,4.03)*	*3.32 (2.87,3.83)*	*2.60 (2.16,3.13)*	*2.49 (2.06,3.01)*

Data were presented with OR and 95% CI; *COR* crude odds ratio, *AOR* adjusted odds ratio, *CI* confidence interval, *VD* vaginal delivery, *CS* Cesarean section

Italic value indicates statistical significance (*P* < 0.05)

**Fig. 1:**
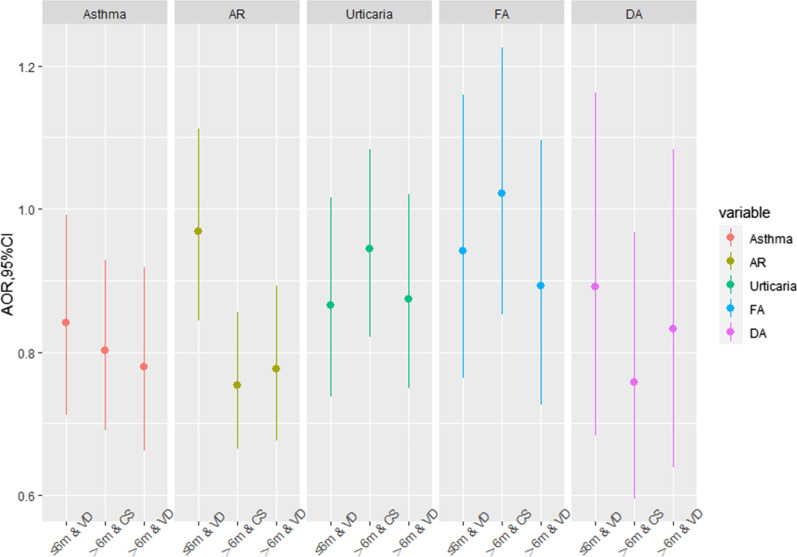
Combined effects of breastfeeding duration & mode of delivery on childhood asthma and allergic diseases. *AR* allergic rhinitis, *FA* food allergy, *DA* drug allergy; Reference group: ≤ 6 m & CS. The confounders adjusted in the model included child’s sex, age, SES, only one child in the household and family history of allergy

**Fig. 2:**
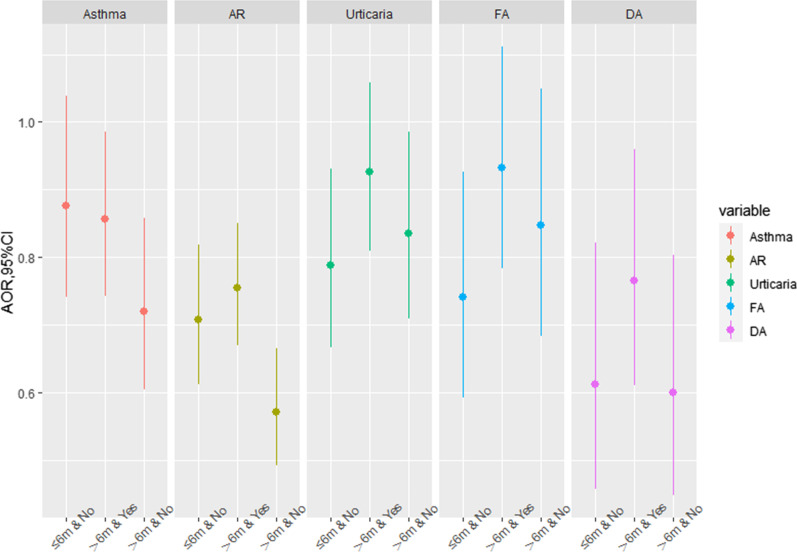
Combined effects of breastfeeding duration & only one child on childhood asthma and allergic diseases. *AR* allergic rhinitis, *FA* food allergy, *DA* drug allergy; Reference group: ≤ 6 m & Yes. The confounders adjusted in the model included child’s sex, age, SES, mode of delivery and family history of allergy

**Fig. 3:**
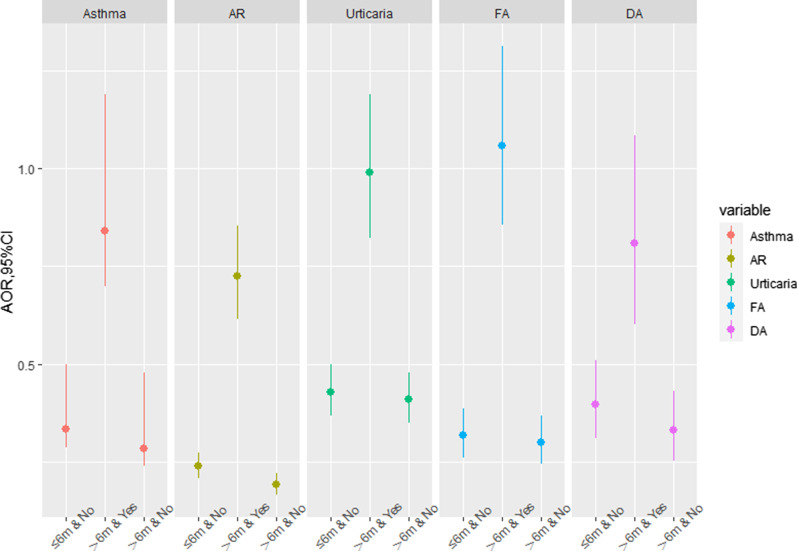
Combined effects of breastfeeding duration & family history of allergy on childhood asthma and allergic diseases. *AR* allergic rhinitis, *FA* food allergy, *DA* drug allergy; Reference group: ≤ 6 m & Yes. The confounders adjusted in the model included child’s sex, age, SES, only one child in the household and mode of delivery

Longer breastfeeding duration (> 6 months) might reduce the associations of delivery mode, family history of allergy, and only one child in the household with childhood asthma and allergic diseases (Figs. 1, 2, 3). For childhood asthma, AR and DA, the AOR in the group of CS and breastfeeding duration > 6 months (0.80, 95% CI: 0.69, 0.93; 0.75, 95% CI: 0.66, 0.86; and 0.76, 95% CI: 0.59, 0.97, respectively) was lower than that in the group of VD and breastfeeding duration ≤ 6 months (0.84, 95% CI: 0.71, 0.99; 0.97, 95% CI: 0.84, 1.11; and 0.89, 95% CI: 0.68, 1.16, respectively) compared to the reference group of CS and breastfeeding duration ≤ 6 months (Fig. 1). It indicates that breastfeeding duration > 6 months might decrease the AOR of CS on childhood asthma, AR and DA. The AOR in the group of VD and breastfeeding duration > 6 months was lowest (0.78, 95% CI: 0.66, 0.92) for childhood asthma.

Similarly, the AOR in the group of breastfeeding duration > 6 months and more than one child in the household was lowest for childhood asthma (0.72, 95% CI: 0.61, 0.86), AR (0.57, 95% CI: 0.49, 0.67) and DA (0.60, 95% CI: 0.45, 0.80) compared to the reference group of breastfeeding duration ≤ 6 months and only one child in the household (Fig. 2).

Furthermore, the AOR in the group of breastfeeding duration > 6 months and without family history of allergy was lowest for childhood asthma (0.28, 95% CI: 0.24, 0.33), AR (0.19, 95% CI: 0.17, 0.22), urticarial (0.41, 95% CI: 0.35, 0.48), FA (0.30, 95% CI: 0.24, 0.37) and DA (0.33, 95% CI: 0.25, 0.43) compared with the reference group of breastfeeding duration ≤ 6 months and having family history of allergy (Fig. 3).

## Discussion

In this population-based cross-sectional study, we found that male sex, high SES, CS delivery, only one child in the household and having family history of allergy were associated with increased OR of childhood asthma and allergic diseases. The longer breastfeeding duration (> 6 months) was inversely associated with childhood asthma and allergic diseases, and could attenuate the effects of CS delivery, only one child in the household and having family history of allergy on childhood asthma and allergic diseases.

Our findings are consistent with most previous studies in which CS delivery, only one child in the household and formula feeding were associated with an increased risk of allergic diseases among children [6–8, 12–18]. Recently, a systematic review and meta-analysis reported that male sex, short duration of breastfeeding and having siblings were risk factors of early transient wheezing among children aged 3–18 years [19]. Our findings also show that male sex and short duration of breastfeeding were associated with increased OR of childhood asthma and allergic diseases, but only one child (having no siblings) in the household was inversely associated with childhood asthma and allergic diseases. In addition, Chu et al. [6] reported that breastfeeding attenuated the impacts of CS on childhood asthma and AR. However, Liao et al. [9] suggest that the association of CS with developing childhood allergy was not modified by breastfeeding duration. The results from this large epidemiological study provide supportive evidence that breastfeeding duration modifies the association between CS and childhood allergy. The inconsistency between studies may be due to the differences in geographic locations, study designs and population characteristics.

Hygiene or old friends hypotheses involved gut microbiota exposure might partly explain the associations of CS, only one child in the household, and breastfeeding with childhood asthma and allergic diseases [20]. A meta-analysis reported an increase in the risk of childhood asthma after CS (OR = 1.20, 95% CI: 1.14, 12.6) [21]. Birth by CS causes development of the gut microbiota to be delayed and to take an unusual course [22]. Only one child, without siblings, also modulates the gut microbiota, leading to allergic disorders [23, 24]. However, breastfeeding could prevent allergy through regulating infant gut barrier function and microbiota [25], which may explain why longer breastfeeding duration attenuated the AOR of CS and only one child in the household on childhood asthma and allergic diseases. CS might boost inflammatory responses, affect bronchial epithelial barrier function, or associate with the metabolic syndrome, or both. In turn, breastfeeding could strengthen bronchial epithelial barrier function and boost innate immunity and regulatory immune responses [26].

Similar to previous studies [2, 17, 19, 27–29], we found that family history of allergy was strongly associated with increased OR of childhood asthma and allergic diseases. Longer breastfeeding duration could reduce these ORs. The World Health Organization issued a recommendation that mothers should breastfeed their children exclusively for 6 months at least [30]. Exclusive breastfeeding is the internationally preferred method of feeding babies during their first 6 months of life, and is recognized as one of the most natural and best forms of preventive medicine. Our findings provide some evidence for supporting this idea – viz., exclusive breastfeeding for at least 6 months and continued breastfeeding for longer will likely reduce the occurrence of childhood allergy.

There are three major strengths in this study. First, a representative sample was obtained through a stringent multi-stage and multi-strata random sampling approach. Second, this large scale population-based cross-sectional study achieved a high response rate (> 95%), so selection bias was probably minimal. Third, a wide range of childhood allergic diseases including asthma, AR, urticaria, FA and DA were considered in this study to explore their associations with a range of neonatal and familial risk factors.

This study also has several limitations. First, all the information was obtained from questionnaires, and some bias (e.g., recall and measurement bias) was inevitable to some extent. For example, the diagnoses of childhood allergy were recalled but were not made by objective allergic testing. The information on breastfeeding duration was not perfect because it was collected retrospectively rather than prospectively. Second, the nature and severity of childhood asthma and allergic diseases was not measured in this study, and therefore, the determinants of mild and severe cases cannot be distinguished. Finally, a causal relationship cannot be established due to the cross-sectional study design. However, the independent variables included in the multivariable model (e.g., child’s sex, CS, only one child in the household and family history of allergy) were significantly earlier than the diagnosis of childhood asthma and allergic diseases, so a certain temporal relationship exists.

Despite these limitations, the results from this study have demonstrated the contemporary prevalence of asthma and allergic diseases among children aged 6–11 years and their associations with CS, only one child in the household, family history of allergy and breastfeeding duration. Longer breastfeeding duration appeared to decrease the effects of the neonatal and familial factors on childhood asthma and allergic diseases. Our findings may be used to develop appropriate strategies to prevent and control childhood asthma and allergic diseases if they are confirmed by further research.

## Conclusions

Among primary school children aged 6–11 years in Shanghai, born by CS delivery, only one child in the household, and having family history of allergy were associated with increased OR of childhood asthma and allergic diseases. Longer breastfeeding duration was associated with reduced OR of childhood asthma and allergic diseases, and seemed to attenuate the OR of neonatal and familial factors on childhood allergic diseases. These findings may have important clinical and public health implications.

## Supplementary Information

**Additional file 1: Table S1.** Sensitivity analysis of multivariate logistic regression after adjustment for residential area and whether the address changed after birth.

## Data Availability

The datasets used and/or analysed during the current study are available from the corresponding author on reasonable request.

## Abbreviations

AOR: Adjusted odds ratios
AR: Allergic rhinitis
CI: Confidence interval
CS: Cesarean section
DA: Drug allergy
FA: Food allergy
ISAAC: The International Study of Asthma and Allergies in Childhood
SES: Socioeconomic status
