# Supplementary material for: Breastfeeding duration modified the effects of neonatal and familial risk factors on childhood asthma and allergy: a population-based study
Source: Respir Res. 2021 Feb 6;22:41. doi: 10.1186/s12931-021-01644-9 (PMC7866653; doi:10.1186/s12931-021-01644-9)
Supplement: Supplementary file 1 — Additional file 1: Table S1. Sensitivity analysis of multivariate logistic regression after adjustment for residential area and whether the address changed after birth. [file 12931_2021_1644_MOESM1_ESM.doc]

Supplementary Table 1 Sensitivity analysis of multivariate logistic regression after adjustment for residential area and whether the address changed after birth

|  | **Asthma** | **AR** | **Urticaria** | **FA** | **DA** |
| --- | --- | --- | --- | --- | --- |
| Sex(girls) |  |  |  |  |  |
| Boys | **1.53**  **(1.36, 1.72)** | **1.62**  **(1.47, 1.79)** | **1.17**  **(1.05, 1.30)** | 1.13  (0.98, 1.31) | **1.23**  **(1.02, 1.48)** |
| Age | 0.99  (0.95, 1.02) | 1.03  (1.00, 1.07) | 0.97  (0.93, 1.00) | 0.98  (0.93, 1.02) | **1.09**  **(1.03, 1.16)** |
| Home ownership  (Not have) |  |  |  |  |  |
| have | **1.25**  **(1.07, 1.46)** | **1.70**  **(1.48, 1.95)** | **1.70**  **(1.45, 1.98)** | **1.30**  **(1.06, 1.58)** | 1.19  (0.92, 1.54) |
|  |  |  |  |  |  |
| Mode of delivery  (VD) |  |  |  |  |  |
| CS | 1.11  (0.99, 1.26) | 1.00  (0.91, 1.11) | 1.12  (1.00, 1.25) | 1.10  (0.95, 1.28) | 1.02  (0.84, 1.24) |
| Breastfeeding duration  (≤6m) |  |  |  |  |  |
| ＞6m | **0.85**  **(0.75, 0.95)** | **0.77**  **(0.70, 0.85)** | 0.97  (0.87, 1.08) | 0.99  (0.86, 1.15) | **0.83**  **(0.68, 0.99)** |
| Only child(No) |  |  |  |  |  |
| Yes | **1.16**  **(1.03, 1.32)** | **1.36**  **(1.22,1.51)** | **1.19**  **(1.06, 1.34)** | **1.22**  **(1.04, 1.43)** | **1.45**  **(1.17, 1.80)** |
| Family history  of allergy(No) |  |  |  |  |  |
| Yes | **3.01**  **(2.68, 3.39)** | **3.96**  **(3.58, 4.39)** | **2.35**  **(2.10, 2.63)** | **3.27**  **(2.83, 3.78)** | **2.48**  **(2.05, 3.00)** |

Data were presented with AOR and 95% CI; AOR: adjusted odds ratio; CI: confidence interval; VD: vaginal delivery; CS: Cesarean section.

Bold value indicates statistical significance (P ＜0.05).
